# Lung cellular senescence is independent of aging in a mouse model of COPD/emphysema

**DOI:** 10.1038/s41598-018-27209-3

**Published:** 2018-06-13

**Authors:** Kahkashan Rashid, Isaac K. Sundar, Janice Gerloff, Dongmei Li, Irfan Rahman

**Affiliations:** 1 Department of Environmental Medicine, University of Rochester Medical Center, Rochester, NY USA; 2 Department of Clinical & Translational Research, University of Rochester Medical Center, Rochester, NY USA

## Abstract

Cigarette smoke (CS) induces lung cellular senescence that plays an important role in the pathogenesis of chronic obstructive pulmonary disease (COPD). How aging influences cellular senescence and other molecular hallmarks, and increases the risk of CS-induced damage remains unknown. We hypothesized that aging-associated changes in lungs worsen the COPD/emphysema by CS exposure. Younger and older groups of C57BL/6J mice were exposed to chronic CS for 6 months with respective age-matched air-exposed controls. CS caused a decline in lung function and affected the lung structure of both groups of mice. No alterations were observed in the induction of inflammatory mediators between the air-exposed younger and older controls, but aging increased the severity of CS-induced lung inflammation. Aging *per se* increased lung cellular senescence and significant changes in damage-associated molecular patterns marker S100A8. Gene transcript analysis using the nanoString nCounter showed a significant upregulation of key pro-senescence targets by CS (*Mmp12, Ccl2, Cdkn2a, Tert, Wrn*, and *Bub1b*). Aging independently influenced lung function and structure, as well as increased susceptibility to CS-induced inflammation in emphysema, but had a negligible effect on cellular senescence. Thus, aging solely does not contribute to the induction of cellular senescence by CS in a mouse model of COPD/emphysema.

## Introduction

Aging is defined as a result of the progressive breakdown of tissues and organs, an imbalance in physiological processes, and a reduced response to environmental challenges^1^. Most parts of the body including the lungs experience progressive damage with aging as well as impaired function^2^. Lung aging is associated with loss of elasticity, a decrease in pulmonary function, loss of structural integrity, and an increase in inflammation which are among the key characteristics of chronic obstructive pulmonary disease (COPD). COPD is the third leading cause of chronic morbidity and mortality on a global scale. Growing evidence suggest that age-associated structural and functional alterations enhance pathogenetic susceptibility to COPD^3,4^.

Along with other toxic gases, the most common etiological factor that develops COPD is cigarette smoke (CS) which results in several pathophysiological changes in the lung. Small airway remodeling, mucus hypersecretion, enlargement in alveolar space, chronic inflammation of airways, and accelerated premature lung aging (destruction of lung parenchymal cells and decline in lung function) are the most common hallmarks of aging^2,4,5^. Recent reports suggest that CS induces oxidative stress-mediated DNA damage and triggers cellular senescence in the lungs^6,7^. Cellular senescence is a process of complete and permanent cell cycle arrest^8^. Earlier evidence show its contribution to different biological processes, such as embryonic development, wound healing, tissue regeneration and aids in cancer prevention^9,10^. On the other hand, the accumulation of metabolically active senescent cells in tissues during aging impairs tissue repair and function. Pro-inflammatory mediators are secreted which give rise to a phenomenon known as senescence-associated secretory phenotype (SASP)^11,12^. Senescent cells increase the damage of neighboring cells by virtue of their SASP phenotype^13–15^. Previous reports proposed a network of cellular senescence, inflammatory response, and premature lung aging in the pathogenesis of COPD^4^.

CS exposure and aging play a vital complementary role in the pathogenesis of COPD. We hypothesize that chronic CS exposure leads to COPD/emphysema in mice (younger and older), lung aging-associated changes which could directly result in COPD/emphysema-like phenotype in an age-dependent manner (i.e. in older mice), and that CS exposure aggravates COPD/emphysema in older mice as compared to their younger counterparts. We tested this hypothesis by exposing the mice of different ages (relatively younger mice: eight months of age; and relatively older mice: seventeen months of age) to chronic CS (6 months). We then measured various biological (physiological and anatomical) markers associated with aging, lung function, lung anatomy, inflammatory cellular influx, pro-inflammatory mediators, and the expression of different genes associated with cellular senescence and other molecular hallmarks of lung damage.

## Materials and Methods

Unless otherwise stated, all biochemical reagents used in this study were purchased from Sigma Chemicals (St. Louis, MO, USA). The antibodies listed for mouse samples were of commercial grades and validated by manufacturers based on their data sheets.

### Ethical approval: Animal study protocol and institutional biosafety approvals

All animal protocols and procedures described in this study were approved by the University Committee on Animal Research Committee of the University of Rochester, Rochester, NY. All experiments performed in this study were approved and accordance with the University of Rochester Institutional Biosafety Committee.

### Mice and exposure to chronic cigarette smoke

C57BL/6 J mice (Jackson Laboratory, Bar Harbor, ME, USA) were bred and maintained with a 12 h light/dark cycle in the vivarium facility of the University of Rochester. Male and female mice with different ages (8–10 months and 17–18 months) were exposed to cigarette smoke generated by research grade cigarettes (3R4F) according to the Federal Trade Commission protocol (1 puff/min of 2-s duration and 35-ml volume for a total of 8 puffs at a flow rate of 1.05 L/min) with a Baumgartner-Jaeger CSM2072i automatic CS generating machine (CH Technologies, Westwood, NJ)^16,17^. The mainstream smoke exposure was performed at a concentration of ~250–300 mg/m^3^ total particulate matter (TPM) by adjusting the flow rate of the diluted medical air, and the level of carbon monoxide in the chamber was measured at ~350 ppm^16^. Mice which were 14–16 months old at the end of the experiment were referred to as younger mice compared to mice 23–24 months old which were termed as older mice. The same protocol of CS exposure was followed for both the younger and older mice. Control mice were exposed to filtered air and other mice were exposed to chronic CS for 5 h per day, 5 d per week for 6 months in an identical chamber. After 24 h of the last exposure, all the mice were sacrificed and their tissues were used for performing different experiments.

### Collection of bronchoalveolar lavage (BAL)

Mice were injected with a single intraperitoneal dose (90 mg/kg body weight) of pentobarbiturate (Abbott Laboratories, IL) and sacrificed by exsanguination. The heart and lungs were removed *en bloc*. A solution of sodium chloride (0.9%) was used to lavage lungs for three times^18,19^. The lavage fluid was centrifuged, and the cell-free supernatants were frozen at −80 °C for later analysis. The BAL cell pellet was resuspended in 1 ml of 0.9% sodium chloride, and stained by acridine orange propidium iodide (AO/PI) stain to determine the total cell counts/ml using cellometer.

### Labeling of BAL cells and differential cell count using flow cytometry

Cell-type specific monoclonal antibodies were used for flow cytometric analysis of inflammatory immune cells. Briefly, 2.0 × 10^5^ to 4.0 × 10^5^ cells were stained with 1x PBS using cell-type specific markers for 30 min., then washed and re-suspended in 0.1 ml of 1x PBS for analysis. Cell-specific markers such as LY6B.2 Alexa Fluor 488-conjugated antibody for neutrophils (Novus Biologicals Cat# NBP213077AF488) CD8a PE-cy5-conjugated antibody for T-lymphocytes (BD Biosciences 553034) and F4/80 PE-conjugated antibody for macrophages (BioLegend Cat #123109) were used. Flow cytometry data acquisition was performed on a BD Accuri flow cytometer (BD Accuri C6 software) and analyzed using FlowJo software.

### Lung morphometry

Non-lavaged mouse lungs were inflated with 1% low melting agarose at a pressure of 25 cmH_2_O. Formalin (10%) was used to fix those lungs and fixed lungs were dehydrated and embedded in paraffin. Midsagittal sections of lungs of 4 µm were made using a rotary microtome (MICROM International GmbH, Walldorf, Germany). Hematoxylin and eosin (H&E) staining was performed on the lung midsagittal sections to determine mean linear intercept (Lm) of airspace using the MetaMorph software (Molecular Devices)^18,19^. Ten randomly selected ×100 fields per slide were photographed, and the images were manually threshold. Each stained slides were analyzed in a blinded fashion for the presence of alveolar space enlargement, destruction of alveolar wall, and infiltration of inflammatory cells.

### Protein extraction and quantification

One lobe of the lung tissue weighed ~48 mg was homogenized (Pro 200 homogenizer, at maximum speed, 5th gear for 40 seconds) in 0.48 ml of ice-cold 1X cell lysis buffer (5 mM CHAPS, 40 mM citric acid, 40 mM sodium phosphate, 0.5 mM benzamidine, and 0.25 mM PMSF, pH 6.0) containing complete protease inhibitor cocktail (Sigma). The tissue homogenate was then incubated on ice for 1 h to allow the cells to lyse completely. The lysates were centrifuged at 12,000 g for 10 min at 4 °C to separate the protein fraction from the cell/tissue debris and the supernatants containing protein were transferred into the new tubes for measuring SA-β-gal activity and some supernatants were aliquoted to store at −80 °C for performing Western blot. Protein content from the samples was estimated by bicinchoninic acid (BCA) colorimetric assay (Thermo Scientific, Rockford, IL) using BSA as a standard.

### Measurement of SA-β-gal activity

Senescence Associated-β-galactosidase (SA-β-gal) activity was measured quantitatively using cellular senescence activity assay kit (Enzo Life Sciences, Farmingdale, NY) according to the manufacturer’s protocol. Quantitative measurement of SA-β-gal activity depends on the rate of conversion of 4-methylumbelliferyl-β-D-galactopyranoside (MUG) to the fluorescent hydrolysis product 4-methylumbelliferone (4-MU) at pH 6.0, as described previously^20,21^. In brief, protein fraction containing supernatants were mixed with 2X reaction buffer containing the substrate (40 mM citric acid, 40 mM sodium phosphate, 300 mM NaCl, 10 mM β-mercaptoethanol, and 4 mM MgCl2 [pH 6.0] with 1.7 mM MUG) and incubated at 37 °C for 3 hours. Finally, 50 μl of reaction mixture was added to 200 μl of stop solution in a 96-well fluorescent plate and fluorescence was measured using a SpectrumMax M5 plate reader (Molecular Devices) at 360 nm (excitation) and 465 nm (emission). Normalized SA-β-gal activity was expressed as observed fluorescence divided by total protein in milligram/ml.

### Immunoblot analysis

Twenty-five micrograms of proteins that were extracted from whole lungs were subjected to electrophoresis either on 14% sodium dodecyl sulfate (SDS)-polyacrylamide gel or 4–15% gradient (SDS)-polyacrylamidegel (Bio-Rad). Those gels were transferred onto nitrocellulose membrane (Amersham, Arlington Heights, IL, USA) and were blocked for 1 h at room temperature with 5% non-fat dry milk in phosphate-buffered saline containing 0.1% Tween 20. Membranes were then probed with specific primary antibodies like anti-p16 ARC (ab51243, Abcam), anti-GAPDH or anti-actin (sc-365062, sc-1616, Santa Cruz, CA) (1:1000 dilution) and incubated overnight at 4 °C. After six 5-minute washing steps, the membranes were probed with suitable secondary anti-rabbit, or anti-mouse, or anti-goat antibody (1:10,000 dilution in 5% milk) linked to horseradish peroxidase for 1 h, and detected using the enhanced chemiluminescence method (Perkin Elmer, Waltham, MA) and images were taken with Bio-Rad ChemiDoc MP, Imaging system. Equal loading of the samples was determined by reprobing membranes with GAPDH/Actin. Band intensity was calculated by densitometry analysis and expressed as fold change relative to corresponding loading control.

### Assessment of pro-inflammatory mediators in BAL fluid and plasma

The level of proinflammatory mediators (such as MCP-1, KC, MIP-1α, IL-12p40, and G-CSF) in BAL fluid (50 μL) was measured by Luminex multiplex assay using Bio-Plex Pro mouse cytokine immunoassay kit (Bio-Rad, Hercules, CA) according to the manufacturer’s instructions^16,22^. The levels were expressed as pg/ml for BAL fluid.

Mouse cytokine array analysis was performed for the detection of the relative expression of 40 different mouse cytokines. We used pooled BAL fluid samples (n = 4–5 mice/group) from air and CS-exposed mice for probing the arrays following the manufacturer’s instructions (R&D Systems, Inc. Minneapolis, MN).

The levels of damage-associated molecular patterns (DAMPs) markers (soluble RAGE, and its ligand S100A8 and HMGB1) in BAL fluid and (S100A8) plasma samples were determined using corresponding enzyme-linked immunosorbent assay kits according to manufacturer’s instructions (R&D Systems, Inc. Minneapolis, MN).

### Senescence and aging-related gene expression panel by nanoString

RNA isolated from lung tissues of chronic air and CS-exposed younger and older mice using a Qiagen RNeasy Mini Kit (Qiagen). Isolated RNA was processed through the nanoString nCounter system and given to the nanoString Technologies (Seattle, WA) for analysis. The concentration of submitted RNA was upto 150–300 ng. The code set was custom designed for us by the company specific for 41 different mouse cellular senescence genes including five housekeeping genes (*Gapdh*, *Hprt1*, *Rpl19*, *Tbp*, and *Tubb5*). nanoString mRNA counts were normalized, log2 transformed for differential analysis using linear models in the limma package (R/Bioconductor). Comparison between different experimental groups (younger-Air vs. younger-CS, older-Air vs. older-CS, younger-Air vs. older-Air and younger-CS vs. older-CS) were performed using linear contrast model, moderated t statistics were used to determine the differences in gene expression levels with empirical Bayes approach. The Benjamini-Hochberg procedure was further used to adjust the p-values to control the false discovery rate at 5%. Normalized nanoString mRNA counts were used to represent selected genes from the cellular senescence and aging panel that was differentially expressed among younger and older chronic air and CS-exposed mouse lungs. Sequences for custom probe design used in this study for mouse cellular senescence panel were based on the nanoString technology (Supplemental Table 1).

### Statistical analysis

Statistical analysis of significance was calculated using unpaired Student’s *t*-test. The probability of significance compared to control was based on 2-tail *t*-tests. Statistical analysis of significance was calculated using two-way Analysis of Variance (ANOVA) for multi-group comparisons (Tukey’s multiple comparison tests) using GraphPad Prism 7 (La Jolla, CA). The results were shown as the mean ± SEM. ^*^P < 0.05, ^**^P < 0.01, ^***^P < 0.001, ^#^*P* < 0.05, ^##^*P* < 0.01, ^###^*P* < 0.001 which were considered as statistically significant.

## Results

### Aging contributes towards the augmentation of cellular SA-β-gal activity, but the increase in CS-induced cellular senescence is independent of aging

**Figure 1:**
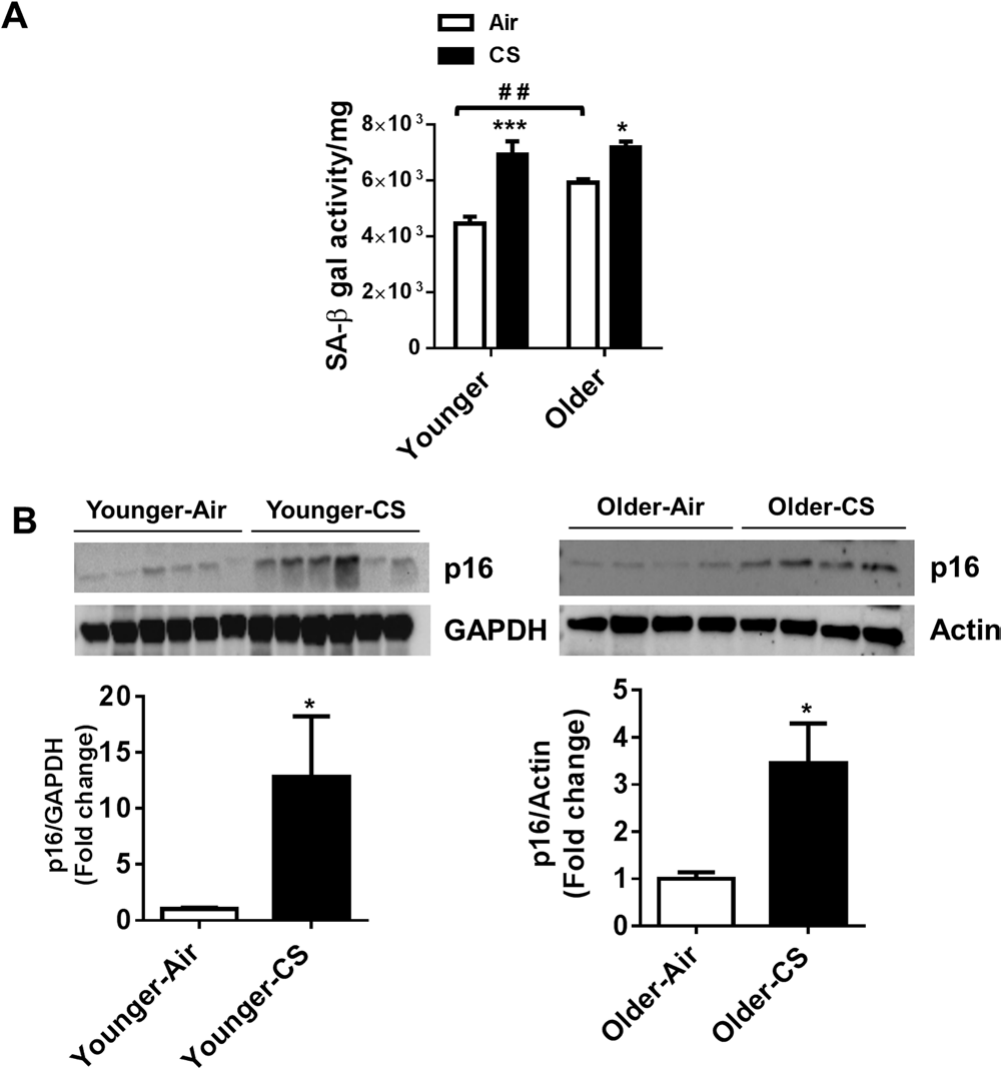
Aging contributes on the augmentation of cellular SA-β-gal activity, but increase in CS induced cellular senescence is independent of aging. Younger and older mice were exposed to CS for 6 months (**A**) SA-β-gal activity (after 3 hours of incubation) and (**B**) expression of cellular senescence marker p16 was measured in lung homogenates using SA-β-gal activity assay kit and immunoblot analysis respectively. GAPDH/Actin was used as loading controls. The band intensity was measured by densitometry and data were shown as fold change relative to respective control group. Full Gels/blots with bands (unedited/uncropped electrophoretic gels/blots) obtained from air- and CS-exposed younger and older samples from the same experiments were processed in parallel are shown (see Supplementary information as Suppl Fig. 1). Data are shown as mean ± SEM (n = 6–12/group). For SA-β-gal activity significance determined using 2-way ANOVA followed by Tukey’s multiple comparisons test and for immunoblot analysis unpaired t-test was performed. *P < 0.05, ***P < 0.001, respective air-exposed control groups; ^##^*P* < 0.01 vs. younger air.

SA-β-gal activity (lysosomal) and increased expression of p16 are considered to be valid markers of cellular senescence. SA-β-gal activity was measured in lung homogenates of air and CS-exposed younger and older mice for *in vitro* incubation periods lasting for 3 h. Older air-exposed mice showed a considerable increase in SA-β-gal activity as compared with younger air-exposed mice after 3 h. A significant increase in SA-β-gal activity was also observed in both younger and older mice exposed to CS (Fig. 1A). Additionally, no significant change in SA-β-gal activity was found in CS-exposed younger mice compared to older mice (Fig. 1A). Along with SA-β-gal activity, an increase in the expression of cyclin-dependent kinase inhibitor p16 was also found in the CS-exposed younger and older mice compared to their respective air-exposed controls (Fig. 1B). Collectively, these results show that the characteristic of aging itself is involved in lung cellular senescence, but does not increase the susceptibility of CS-induced lung cellular senescence in a mouse model of COPD.

Lipofuscin pigments (a nondegradable oxidation product of lipids, proteins and metals from lysosomal or membranous degradation), which accumulates in post-mitotic cells (mainly in fibroblasts), can be detected when released and oxidized or peroxidation process. It’s a non-specific marker of aging tissues. Detection of lipofuscin produces false-positive results with oxidized lipids. We attempted to determine lipofuscin by Sudan Black B staining in lung sections^23^ and ELISA methods in lung homogenates using the ELISA kit from MyBioSource (Cat #MBS721717). However, we were unable to clearly detect the pigments in airway and alveolar regions, though some dark blue-black staining was seen in macrophages (data not shown) which were infiltrated in the airways (in older CS group). Similarly, the ELISA assay produced confounding results without any appreciable change in lipofuscin levels in all the groups (data not shown). This suggests that lipofuscin is a non-specific marker of cellular senescence which is not altered by CS and/or aging at least in the lung.

### Chronic CS exposure and aging contributes to decline in lung function

**Figure 2:**
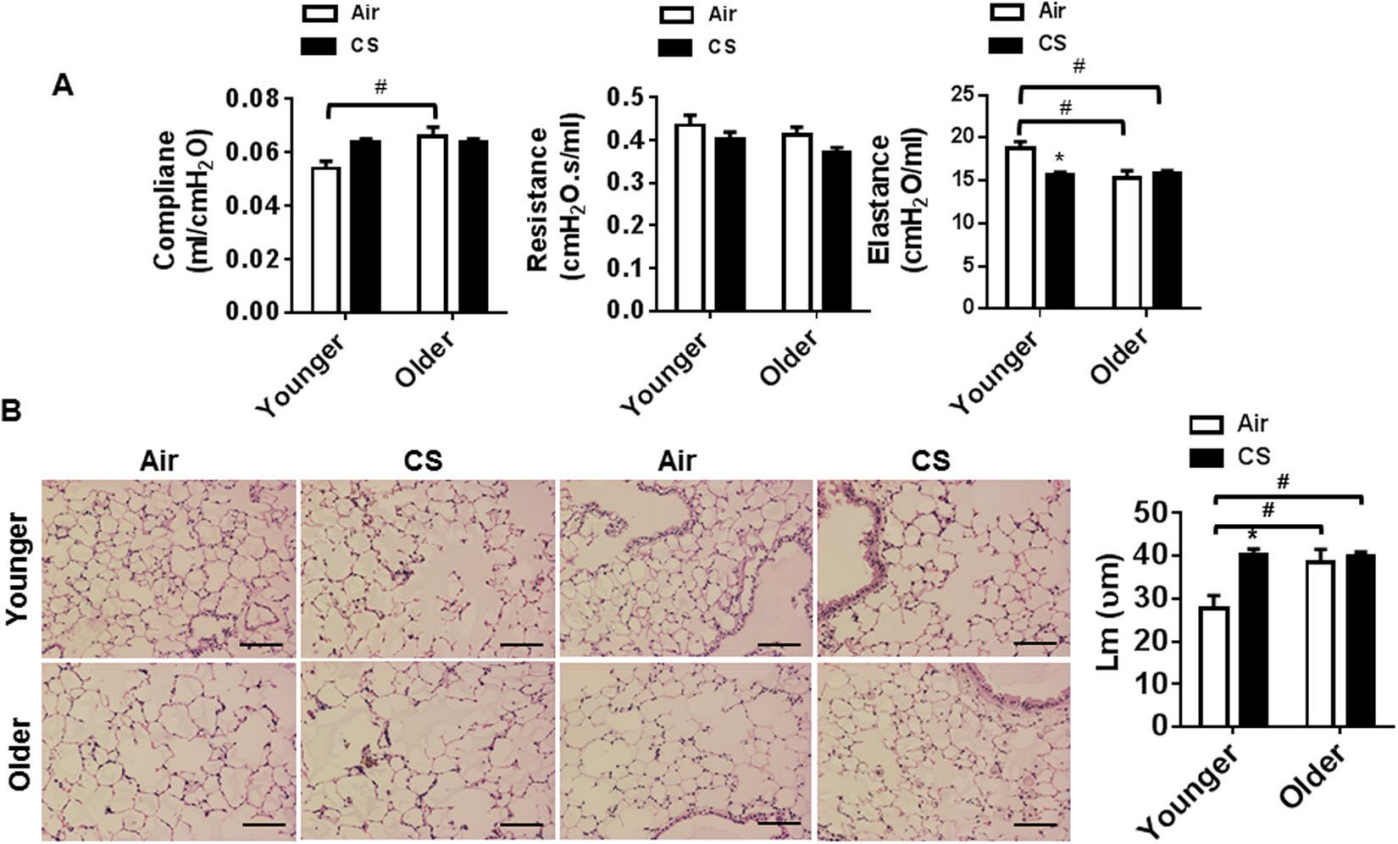
Chronic CS exposure impaired lung function and airspace enlargement in an age-dependent manner. (**A**) Exposure to chronic CS for 6 months altered different parameters related to lung function (compliance, resistance and elastance) in younger and older mice. (**B**) Lung morphometry analysis (Lm) was measured in paraffin-embedded lung sections stained with H&E by MetaMorph^TM^ (Molecular Devices). Original magnification, x200; Scale bar (100 μm). Data are shown as mean ± SEM (n = 4–6/group). Significance determined using 2-way ANOVA followed by Tukey’s multiple comparisons test. *P < 0.05, respective air-exposed control groups; ^#^*P* < 0.05, vs. younger air.

Aging is associated with a reduction in lung mechanical properties including lung compliance, resistance, and elastance^24^. A significant increase in lung compliance and a reduction in lung elastance were observed in older air-exposed mice compared to younger air-exposed mice (Fig. 2A). CS exposure led to a modest increase in lung compliance and a significant reduction in lung elastance in younger mice compared to their air-exposed controls. No considerable alteration in lung resistance was found among air and CS-exposed younger and older mice. In addition, no change was observed in lung compliance and elastance between CS-exposed younger and older mice **(**Fig. 2A). We histologically assessed the lungs and measured airspace enlargement (Lm: mean linear intercept) in both the groups. Older air-exposed mice showed enlarged alveolar spaces compared to younger mice. Both age groups exposed to CS exhibited increased inflammatory cell influx (foamy macrophages around airways and alveolar regions) when compared to their respective controls. CS-exposed younger mice and aging itself independently increased Lm to a significant extent compared to the air-exposed younger controls. Whereas exposure to CS in the older mice did not augment Lm, but destruction of the alveolar wall was clearly visible in the older CS-exposed group **(**Fig. 2B). These data suggest that aging affects lung anatomy and takes part in the reduction of lung function due to the incomplete contraction of lung tissue during the expiration phase. Exposure to CS decreases lung function in the younger groups, but affects the pulmonary structure in both the groups.

### Aging increased CS-induced neutrophil influx and pro-inflammatory cytokines in bronchoalveolar lavage (BAL) fluid

**Figure 3:**
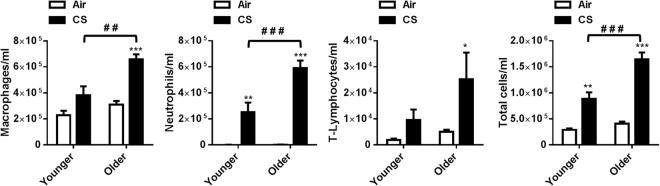
Chronic CS exposure showed age-dependent increase in inflammatory cellular influx in bronchoalveolar lavage (BAL) fluid by CS. Younger and older mice were exposed to CS for 6 months. Differential cell counts were analyzed using BAL fluid and count of inflammatory cells (F4/80^+^ macrophages, CD8a^+^ T-lymphocytes and LY6B.2^+^ neutrophils) was determined by flow cytometry. Cells from BAL fluid were stained with AO/PI to count total number of cells using cellometer. Data are shown as mean ± SEM (n = 5–11/group). Significance determined using 2-way ANOVA followed by Tukey’s multiple comparisons test. *P < 0.05, **P < 0.01, ***P < 0.001, respective air-exposed control groups; ^##^*P* < 0.01, ^###^*P* < 0.001, vs. younger CS.

COPD is a chronic inflammatory lung disease associated with an abnormal inflammatory response induced by CS exposure that is more prevalent in an aged population^4^. In our study, there was no change in the number of inflammatory (macrophages, lymphocytes and neutrophils) and total cells observed between the air-exposed younger and older mice. Chronic CS exposure showed a significant increase in macrophages and total cell counts along with an influx of neutrophils and T-lymphocytes in BAL fluid of both younger and older mice as compared to their respective air-exposed controls (Fig. 3). A considerable increase in the macrophages, neutrophils, lymphocytes and total cell populations were also observed between the CS-exposed groups of younger and older mice (Fig. 3). The increase in total cell count in BAL fluid was due to an increase in the number of inflammatory cellular influx in the older mice as compared to the younger mice exposed to chronic CS.

**Figure 4:**
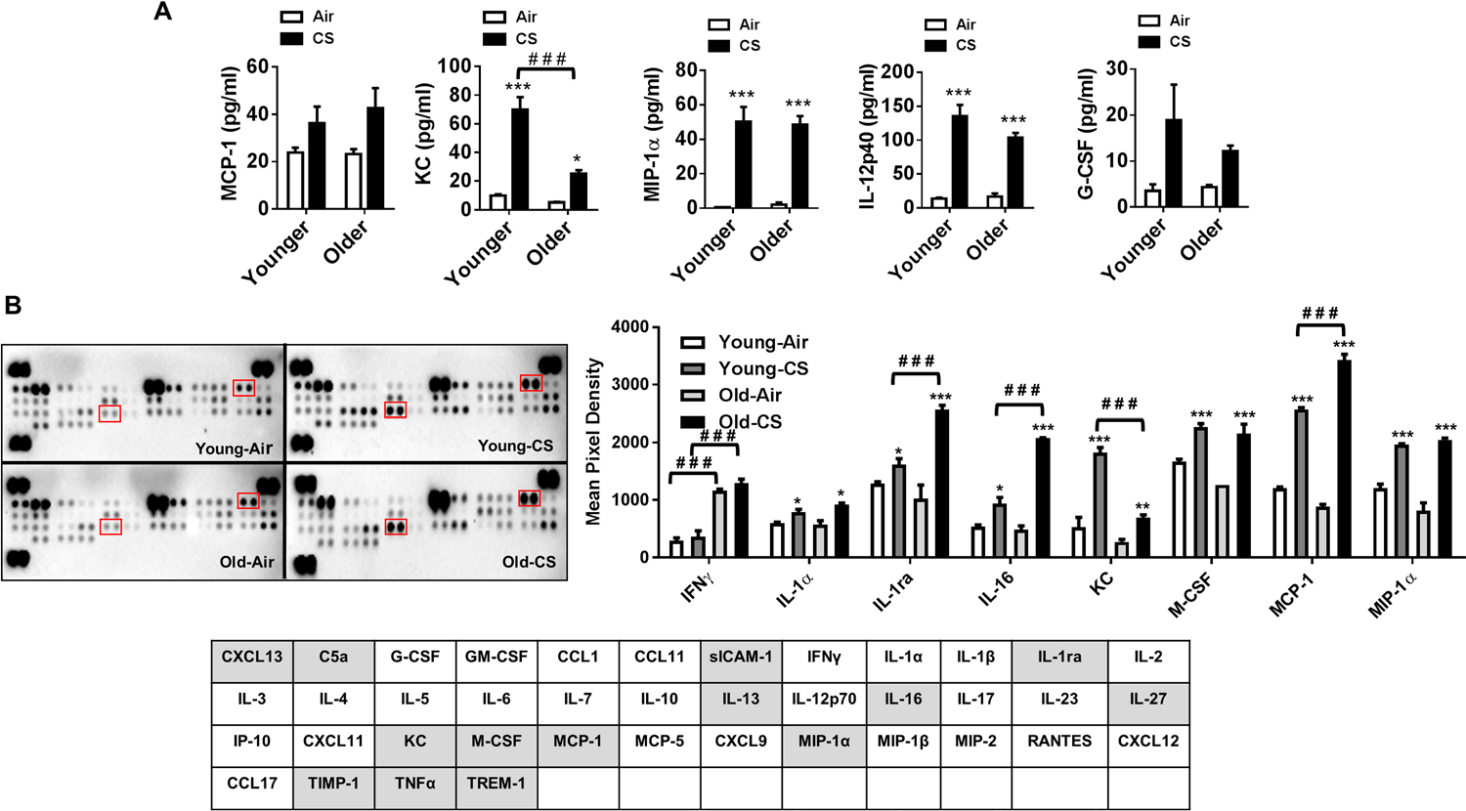
Aging enhanced the level of CS induced proinflammatory cytokines in bronchoalveolar lavage (BAL) fluid. Younger and older mice were exposed to CS for 6 months and BAL fluids were used to determine SASP cytokines. (**A**) The level of proinflammatory cytokines (MCP-1, KC, MIP-1α, IL-12p40 and G-CSF) were measured using luminex multiplex assay. (**B**) Expression of cytokines were also detected from the pooled BAL fluid samples from younger and older mice using mouse cytokine array. Data are shown as mean ± SEM (n = 5–6/group). Significance determined using 2-way ANOVA followed by Tukey’s multiple comparisons test. *P < 0.05, **P < 0.01, ***P < 0.001, respective air-exposed control groups; ^###^*P* < 0.001, vs. younger air or CS.

Evidence suggest that an escalation of aging-associated CS-induced inflammation occurs as a result of the elevated release of cytokines by bronchial epithelial cells^4,25^. Therefore, the levels of specific pro-inflammatory cytokines in BAL fluid were measured by Luminex multiplex assay. Increased in inflammatory cellular influx in CS-exposed younger and older mice was associated with a significant augmentation in secretion of KC, MIP-1α, and IL-12p40 in BAL fluid. A modest increase in the levels of MCP-1 and G-CSF were found in CS-exposed younger and older mice compared to their respective air-exposed controls (Fig. 4A).

Interestingly, cytokine array analysis showed a significant increase in IL-1ra and MCP-1 release in BAL fluid of CS-exposed younger and older mice as compared to their respective air-exposed controls. A comparable increase in IL-1ra and MCP-1 release was also found in CS-exposed older mice compared to CS-exposed younger mice (Fig. 4B). We did not observe any significant change in measured SASP mediators from air-exposed older mice compared to younger mice. Overall, these data revealed that a few SASP mediators were augmented by CS in older mice compared to younger mice, suggesting that both aging and CS-induced SASP together increase the susceptibility to chronic lung diseases.

### Altered DAMPs markers in response to chronic CS exposure

**Figure 5:**
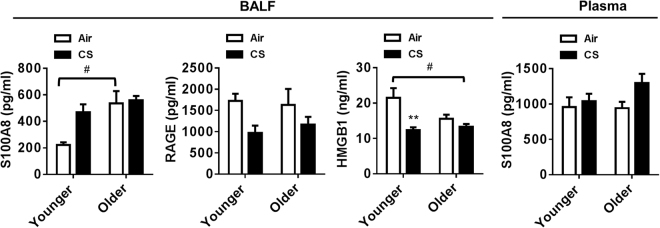
Chronic CS exposure influenced DAMPs markers. Younger and older mice were exposed to chronic CS for 6 months. BAL fluid and plasma were collected from those mice for the measurement of DAMPs markers (S100A8, RAGE and HMGB1) using ELISA. Data are shown as mean ± SEM (n = 5–12/group). Significance determined using 2-way ANOVA followed by Tukey’s multiple comparisons test. **P < 0.01, respective air-exposed control groups; ^#^*P* < 0.05, vs. younger air.

Recent studies suggest that CS exposure induces airway epithelial cell necrosis in COPD patients^26^. This in turn releases DAMPs including high-mobility group box 1 (HMGB1) and S100 calcium binding protein A8 (S100A8) in BAL fluid, serum, and epithelial lining fluid^26,27^. DAMPs bind with different receptors, such as receptor for advanced glycation end product (RAGE) and activate the immune system. An elevation in the level of S100A8 and a significant reduction in HMGB1 were observed in BAL fluid of CS-exposed younger mice as compared to air-exposed controls. No considerable changes in the levels of these two DAMPs ligands were found between CS versus air-exposed older mice, air-exposed younger versus older mice, and CS-exposed younger versus older mice (Fig. 5). In addition, alterations of S100A8 levels measured in plasma did not show significant changes between air and CS-exposed younger and older mice. A trend of reduction in the level of RAGE was found in CS-exposed younger and older mice compared to respective air-exposed controls (Fig. 5). Moreover, aging itself influenced the level of S100A8 in the older mice (Fig. 5). These data suggest that aging contributes in the induction of DAMPs, and CS induced increase in DAMPs in younger mice which was associated with neutrophil-mediated inflammatory response.

### Aging and CS exposure influenced the expression of some cellular senescence-associated genes analyzed by nanoString nCounter analysis

**Figure 6:**
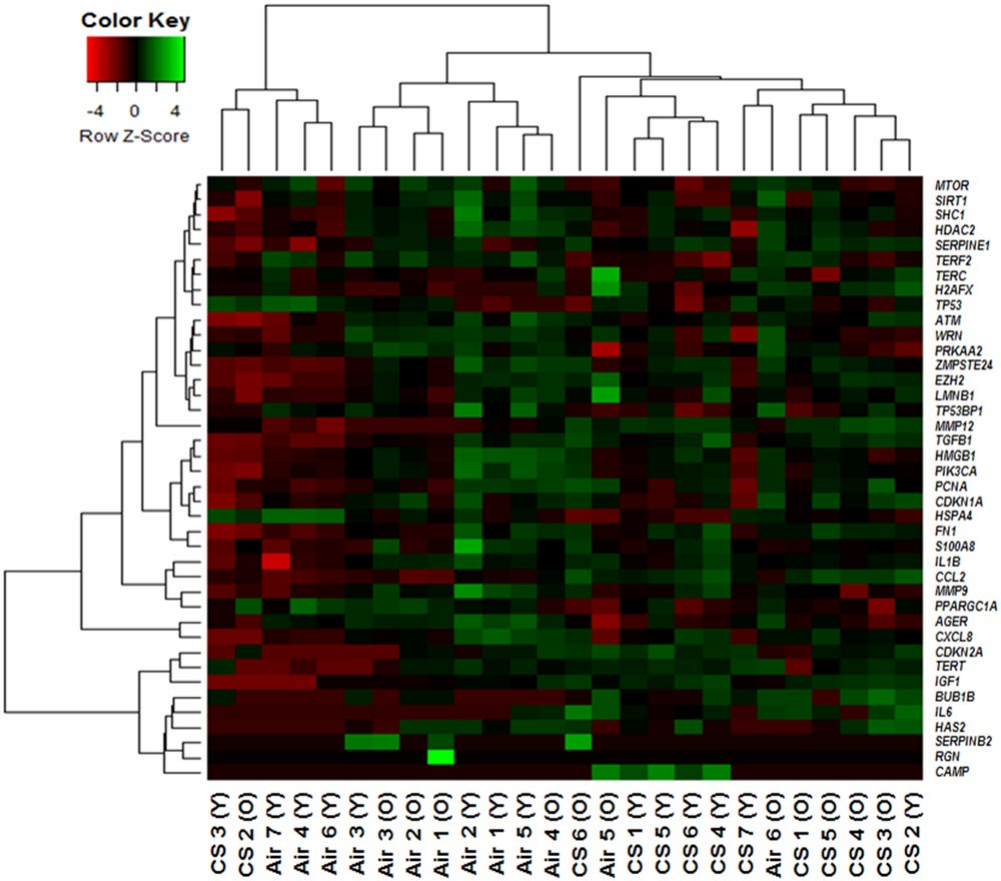
Hierarchical cluster analysis of differentially expressed cellular senescence genes in chronic air- and CS-exposed younger and older mice. Heatmap of normalized expression levels from 41 different cellular senescence genes in mouse lungs analyzed by the nanoString nCounter system. We have omitted 1 gene from the hierarchical cluster analysis since their counts were too low for most of the experimental groups. The green color in the heatmap denotes increased expression and the red color in the heatmap denotes reduced expression of target genes. Target ID and gene list for all the measured cellular senescence gene panels are included (Supplemental Table 1).

Aging may affect the mRNA transcript levels of cellular senescence-associated genes. Further, aging affects the expression of senescence-associated genes in a mouse model of COPD/emphysema. A panel of 41 different mouse cellular senescence genes was designed for differential expression analysis using the nanoString nCounter analysis (Supplemental Table 1). A heatmap was generated using the heatmap.2 function in the gplot package in R. In the heatmap, normalized differentially expressed target genes and sample groups (Younger-Air, Younger-CS, Older-Air, and Older-CS) were included for cluster analysis using the hierarchical clustering method. One gene (Klotho:*Kl*) was removed from the heatmap because of its low count for most of the experimental groups. The heatmap showed the normalized transcript levels from 41 different cellular senescence genes that were analyzed from mouse lungs by the nanoString nCounter analysis system (Fig. 6).

**Figure 7:**
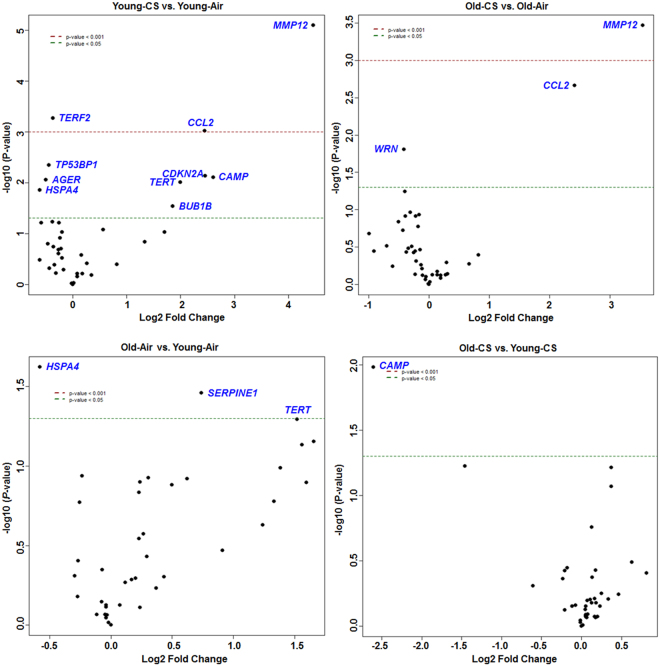
Volcano plots showing differentially expressed cellular senescence genes in younger and older mice exposed to chronic air and CS. nanoString mRNA counts were normalized, log2 transformed for differential analysis using linear models in the limma package (R/Bioconductor). Comparison between different experimental groups were performed using linear contrast model, moderated t statistics was used to determine the differences in gene expression levels with empirical Bayes approach. The y-axis represents the negative log10 *p*-value and x-axis represents the log2 fold change of the comparison. The genes differentially expressed in younger-CS vs. younger-Air, older-CS vs. older-Air, older-Air vs. younger-Air and older-CS vs. younger-CS exposed C57BL/6J mice were marked in blue color. The red and green dotted horizontal lines denote the significance threshold of *P*-values from comparisons *P* < 0.05 and *P* < 0.001. The Benjamini-Hochberg procedure was further used to adjust the *P*-values to control the false discovery rate at 5%.

**Figure 8:**
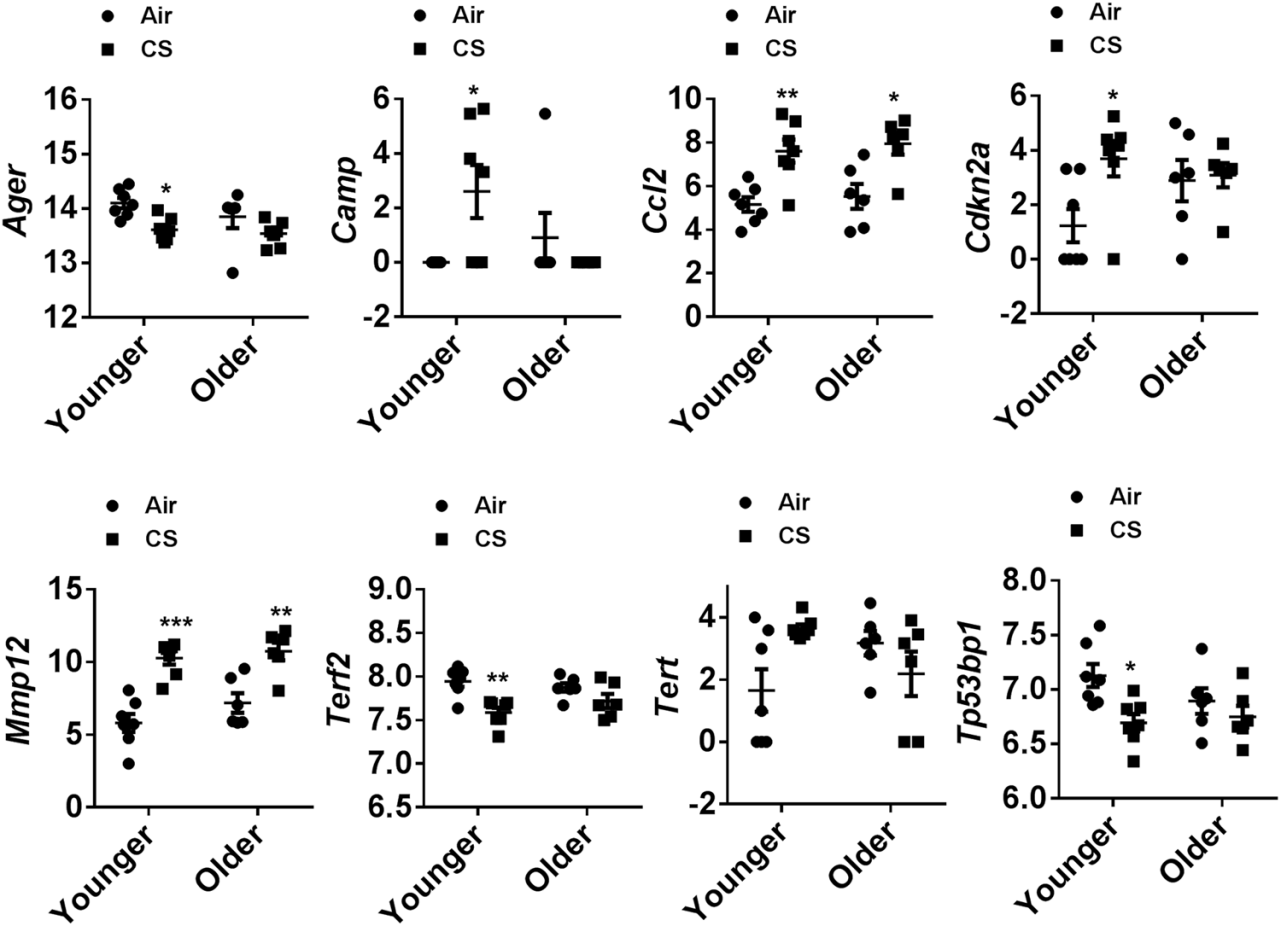
Scatter plots showing differentially expressed cellular senescence genes identified by nanoString in chronic air-and CS-exposed younger and older mice. Normalized nanoString mRNA counts were used to represent selected genes from the cellular senescence panel that was differentially expressed among younger and older chronic air-and CS-exposed mouse lungs. Data are shown as mean ± SEM (*n* = 6–7/group). Statistical significance was determined using 2-way ANOVA followed by Tukey’s multiple comparisons test. *P < 0.05, **P < 0.01, ***P < 0.001 vs. respective air exposed control groups.

**Figure 9:**
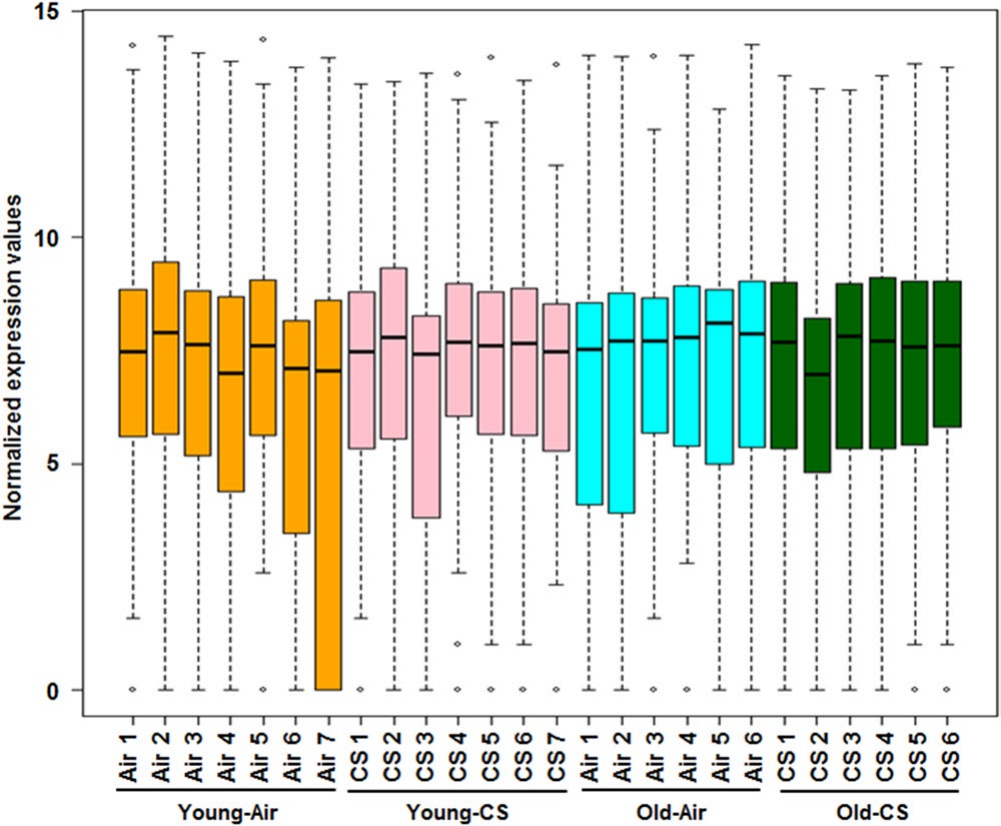
Distribution of normalized expression level of differentially expressed cellular senescence genes. Boxplot shows distribution of normalized expression levels from chronic air and CS-exposed C57BL/6J younger and older mice used for comparison.

Analysis of the normalized mRNA transcript levels among air and CS-exposed younger, as well as older mice, were performed to determine the effects of aging on cellular senescence. A significant increase in the expression of *Mmp12, Ccl2, Cdkn2a, Tert, Camp*, and *Bub1b* and reduction in *Terf2, Tp53bp1*, and *Ager* transcript levels were observed in the CS-exposed younger mice in comparison with their air-exposed controls. Likewise, the older CS-exposed mice showed a considerable increase in *Mmp12* and *Ccl2* transcript levels and a decrease in *Wrn* with respect to air-exposed older mice. In addition, upregulation in the expression of *Serpine1* and *Tert* were significant in the older air-exposed mice as compared with younger air-exposed controls. Finally, analysis of transcript levels in older-CS vs. younger-CS group revealed a reduction only in *Camp* in the older CS mice with respect to younger CS **(**Fig. 7**)**. The genes which play an important role in senescence as well aging in the lung tissue show a considerable alteration in the mRNA transcript levels of *Mmp12, Ccl2, Cdkn2a*, and *Ager* in air-exposed younger and air-exposed older mice with respect to their respective controls (Fig. 8). The nanoString nCounter analysis for Boxplot function in R was used to make a boxplot that shows the normalized distribution of transcript levels from the air or CS-exposed younger and older mice (Fig. 9). Overall, these data depict that aging takes part in the regulation of key cellular senescence-associated genes in mouse model of COPD/emphysema, and chronic CS upregulates the expression of selected senescence pathway genes in the lungs.

## Discussion

Aging and tobacco smoking are both considered to be the major risk factors for the initiation and progression of COPD^25^. It has been proposed that the pathology of COPD is associated with accelerated lung aging^1,6,28,29^. In adults, lungs undergo a progressive decline in its function associated with structural and functional alterations due to increased oxidative stress, inflammatory response, and accumulation of senescent cells in the lungs^30^. CS worsens the process of lung aging and accelerates the progression of stress-induced cellular senescence (SIPS)/SASP as a result of increased oxidative stress, inflammatory response, and DNA damage^31^. We and others have shown that CS exposure in primary lung epithelial cells and fibroblasts undergo cellular senescence^31–34^. However, it has been thought that aging does not contribute to CS-induced COPD/emphysema^5^, whereas certain reports demonstrate that the premature senescence of lung resident cells is involved in the progression of COPD^2,4^.

A number of studies have revealed that smoking adversely affects lung function with increasing age. Smokers who continue to smoke during middle age are more prone for susceptibility to rapid decline in lung function^35–38^. A close associative correlation exists between aging and COPD, but the role and underlying mechanism of aging and cellular senescence in the pathogenesis of COPD, and the impact of chronic CS on the severity of the disease via associated hallmarks of lung damage in an age-dependent manner in younger versus older mice are not known.

**Figure 10:**
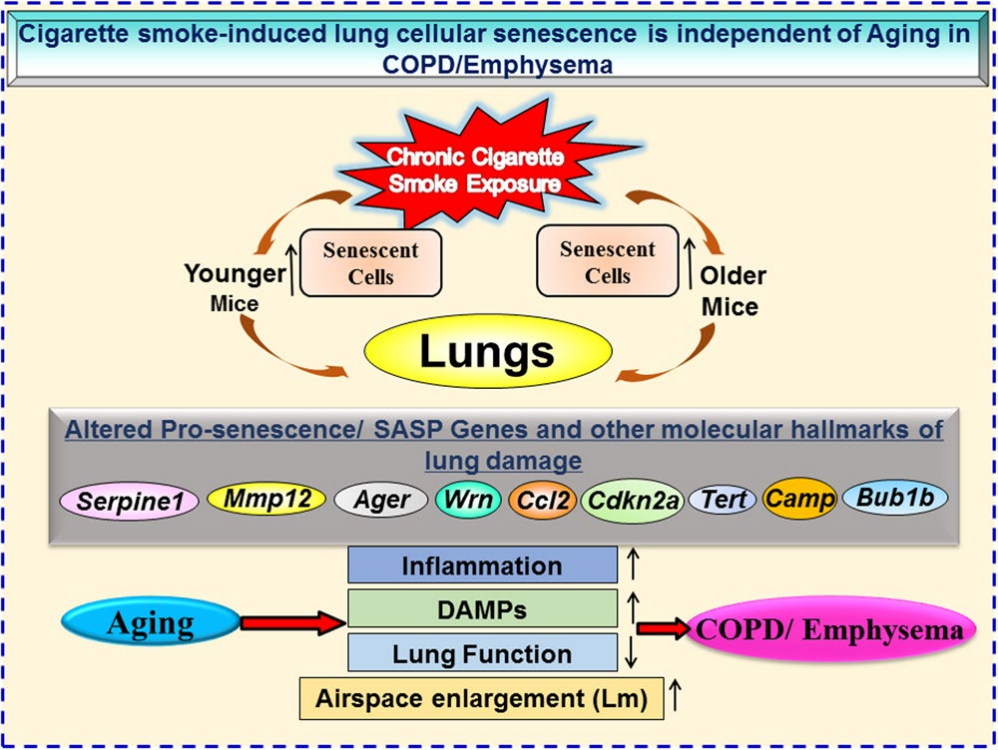
CS-induced lung cellular senescence and other associated molecular hallmarks of lung damage are independent of aging. Younger and older C57BL/6J mice were exposed to chronic CS. Mice of both ages showed lung cellular senescence upon exposure to smoke. CS exposure was associated with alteration in the expression of some of the pro-senescence and SASP related genes. Mice showed increase in inflammation in terms of increased lung influx of inflammatory cells and pro-inflammatory cytokine release. Induction of DAMPs exaggerated lung inflammatory response. Parameters of lung function was affected and led to COPD/emphysema. Chronic CS exposure resulted in cellular senescence-mediated COPD in mice regardless of their age. COPD, Chronic obstructive pulmonary disease; CS, Cigarette smoke and SASP, Senescence associated secretory phenotype.

We investigated the influence of aging and CS exposure on cellular senescence in the pathogenesis of COPD. Mice of different ages (younger and older) were exposed to chronic CS, and SA-β-gal activity was measured in the lungs. Senescence-associated-β-galactosidase (SA-β-gal) is the most common marker of cellular and tissue senescence^39,40^. Chronic CS exposure increased SA-β-gal activity in the lungs of both younger and older mice compared to their respective air-exposed controls. Older air-and CS-exposed mice showed relatively higher SA-β-gal activity compared to the younger air-exposed mice, suggesting an increase of cellular senescence in the lungs as result of physiological aging. However, CS-induced cellular senescence was not different in younger and older mice. Based on these findings, we inferred that aging of mice solely influenced cellular senescence in terms of SA-β-gal activity. We further validated the findings by determining the expression levels of p16^INK4a^, a cyclin-dependent kinase inhibitor, in lungs of both the groups. Senescent cells induce p16^INK4a^ protein abundance and gene expression increases with aging in rodent and human tissues^1,11,21^. Our data confirms the increased expression of p16^INK4a^ in CS-induced cellular senescence in the lungs of younger and older mice. Collectively, these data suggest that normal aging and chronic CS exposure independently induce cellular senescence in the lungs. Thus, the progression of cellular senescence in the mouse model of COPD/emphysema is independent of the aging phenotype **(**Fig. 10**)**.

It has been proposed that aging is associated with a decline in lung function and lung aging may be vulnerable to CS-induced lung pathophysiological alterations. We measured lung compliance, resistance, and elastance to determine the effects of aging and CS exposure on pulmonary function. Lung compliance and elastance show age-dependent alterations in the lungs of older mice. In addition, a similar outcome was observed in younger CS-exposed mice compared to their air-exposed control. We also noticed that aging solely plays a vital role in maintaining lung structure and upon exposure to CS, destruction of alveolar wall was observed in younger mice. Increased alveolar spaces were observed in older air-exposed mice, but destruction of the alveolar wall was not observed in these mice. Upon exposure, CS caused the alveolar walls to breakdown and increased the influx of inflammatory cells in both age groups although no significant alteration was found in the Lm between older air-exposed and older CS-exposed groups. Lung resistance was not affected in younger and older air versus CS, which may in part reflect that structural emphysema may not directly reflect the loss of lung mechanical properties in mice^41^. These findings suggest that aging itself cause a decline in lung function and affect lung structure. Whereas CS exposure does not further augment a decline in lung function in older mice, but does affect lung function in younger CS-exposed mice compared to the air-exposed control.

A connection between inflammatory diseases, such as COPD and aging has been shown in earlier studies^42,43^. Further evidence also demonstrates that older mice are more susceptible to inflammation when exposed to CS, as well as a decreased antioxidant defense mechanism compared to younger mice^5,25,44,45^. We observed heightened macrophages, neutrophil, lymphocyte and total cell counts in BAL fluid of CS-exposed older mice compared to CS-exposed younger mice. An increased inflammatory cellular influx into the lung by CS is demonstrated by elevated levels of neutrophilic chemokines, such as keratinocyte-derived chemokine (KC) and macrophage inflammatory protein 1 alpha (MIP-1α) in BAL fluid of CS-exposed younger and older mice. These data suggest that neutrophilic chemokines play a significant role in the migration of neutrophils into the lungs in younger and older mice exposed to CS, thereby mediating alveolar destruction as a result of releasing a number of damaging factors (serine proteases, elastase, cathepsin-G, proteinase-3, matrix metalloproteinases [e.g. MMP-8 and MMP-9]), and reactive oxygen species^25,46,47^.

We further show CS-induced MCP-1, G-CSF, and IL-12p40 release in BAL fluid of younger and older mice, suggesting both MCP-1 and IL-12p40 may play an important role in macrophages/monocytes chemotaxis, and G-CSF assists in the survival of neutrophils in the respiratory tract^48^. Inflammatory and structural cells (epithelial cells, endothelial cells, and fibroblasts) together contribute to incite inflammation and cellular senescence by secreting pro-inflammatory mediators, proteases, and lipid regulators in the lungs^49^. This phenomenon occurs in senescent cells and is termed as SASP. It induces senescence in the neighboring cells in a paracrine manner by releasing several factors and is considered to be a self-propagating process to enhance inflammation^39,50^. Furthermore, older air-exposed mice did not show increased inflammation due to physiological aging, but an exaggerated inflammatory and injurious responses were ensued when they are exposed to chronic CS.

Damaged cells release DAMPs which trigger an innate immune response by binding with pattern recognition receptors (PRR) either on innate immune cells or on adjacent airway epithelial cells^51^. Activated PRRs (such as RAGE) initiate a signaling cascade that leads to the synthesis of a number of pro-inflammatory cytokines. Some of the cytokines attract cells of the innate immune system (such as neutrophils, macrophages, and dendritic cells) and result in a persistent inflammatory response in COPD^52,53^. Earlier evidence shows a close relationship between the activation of PRR pathways and inflammaging^54–56^. However, it remains unclear how DAMPs markers play a vital role during aging and aging-associated chronic inflammatory lung diseases. Our data reinforces the validity of some previous studies by showing an increase in the level of S100A8, whereas a decrease in HMGB1 in BAL fluid of the younger CS-exposed mice compared to air-exposed control, which is in agreement with the previous findings^57,58^. Moreover, the levels of RAGE in BAL fluid and S100A8 in plasma do not alter significantly in response to CS exposure in younger and older mice. *Ager* is a gene that encodes for RAGE and soluble RAGE inhibits ligation of advance glycation end products (AGEs) with receptor^59^. Prior studies have shown that sRAGE is decreased in BAL fluid of patients with COPD, and AGE accumulation during aging reflect the involvement of AGE-RAGE pathway in the pathophysiology of COPD/emphysema^51,60–62^. Our nanoString mRNA expression data shows decreased expression of *Ager* in CS vs. air-exposed younger mice, whereas no change in the expression of *Ager* was observed between older air vs. older CS-exposed mice. Based on our findings on DAMPs markers, aging alone influenced the release of S100A8 in chronic CS-induced COPD/emphysema. We have shown CS-induced DAMPs may play an important role during the pathogenesis of COPD, which needs further investigation of DAMPs signaling mechanisms during CS exposure both locally in the lung as well as systemically in the circulation.

Finally, we performed nanoString mRNA transcript analysis for selected cellular senescence genes to determine their role in aging and chronic CS exposure-mediated cellular senescence. We observed a significant increase in *Mmp12* in chronic CS-exposed younger and older mice. A previous study suggests that KO of *Mmp12* protects mice from emphysema as it plays an important role in CS-induced airway remodeling^63^. We also noticed that CS exposure induced cellular senescence marker p16 (*Cdkn2a*) in younger mice that were previously assessed by immunoblotting at the protein level and now is confirmed at the mRNA level using the nanoString analysis. Consistent with our earlier data that we obtained from Luminex assay and cytokine array, nanoString analysis showed similar increased expression of *Ccl2* gene (MCP-1) in both younger and older CS-exposed mice. *Bub1b* is a mitotic checkpoint gene which expressed in a cell-cycle dependent manner and helps in a proper segregation of chromosomes. Defects in *Bub1b* are associated with aneuploidy and contribute to the commencement of aging^64^. We found increased expression of mRNA transcript levels of *Bub1b* in CS-exposed younger mice compared to their age-matched air-exposed controls. The reason behind this increase in CS-exposed younger group remains unclear, which requires further study. It has been earlier reported that CS induces senescence on lung tissue (pulmonary epithelial cells and fibroblast) by down-regulating the expression of Werner’s syndrome protein (a member of RecQ helicase family) which is encoded by *Wrn* gene, and accelerates aging in emphysema^65^. In consistent with the previous study, we also observed downregulation of *Wrn* mRNA transcript in older CS vs. older air-exposed mice. Previously, Jiang *et al*. showed that *Serpine1* promotes senescence of alveolar type II cells by inducing p53-p21-Rb cell cycle repression pathway in lung disease^66^. Increased expression of *Serpine1* in older air vs. younger air-exposed mice suggests that *Serpine1* upregulation could mediate cellular senescence of pulmonary cells as the mice age.

Telomere length is one of the important biomarkers of aging and is closely associated with the progression of COPD. Previously, Zhou *et al*. showed that aging influenced telomere length in pulmonary cells, but CS exposure does not exacerbate the condition in aged mice^5^. We measured a number of genes related to telomere length (TL) and DNA damage in the present study. A considerable upregulation in the expression of *Tert* and a significant downregulation in *Terf2* in CS-exposed younger mice were observed. Increased expression of *Tert* was also observed in older air vs. younger air-exposed mice. In addition, a significant reduction in the expression of DNA damage repair associated gene *Tp53bp1* was noticed between younger CS vs. air-exposed mice. These findings suggest that TL-related genes also take part to induce senescence upon exposure to CS in younger mice^67^, but aging neither affected the expression of these genes nor increased the susceptibility to CS-induced cellular senescence. Taken together, these results conclude that aging *per se* influences cellular senescence programming, and CS exposure differentially affects the expression of cellular senescence pathway genes.

Further, we compared 8 months old mice with 17 months old mice to understand the impact of aging on cellular senescence by cigarette smoke. We assigned 8 month old mice as “younger” which actually represent early middle-aged humans^68^, and 17 month old mice as “older”. Though the study has some limitations, it is known that smoking adversely affects lung function with increasing age. Smokers who continue to smoke during middle age are more prone to a rapid decline in lung function^35–38^. Previous studies have compared very young 2 month old mice exposed to 6 months chronic air/CS (8 months old at the end of CS exposure)^18,19^ with older mice^24^, whereas our study is distinct and addresses early middle age vs old age CS-induced lung pathological changes.

Cell-specific SA-β-gal assay would have been more appropriate here to reflect a specific cell population in terms of aging rather than organ aging. We used whole lung homogenate and performed a SA-β-gal assay, which may have obscured the differences in cell-specific senescence by CS exposures in the lung, or it was not augmented by CS in older mice due to inherent age-related lung cellular changes. Another marker of aging, lipofuscin staining and ELISA data in the lung showed no appreciable detection and/or confounding results suggesting that lipofuscin (lipid fragments from lysosomal or membranous degradation) is not a specific marker of lung cellular senescence. This may be due to lung structural destruction during aging and CS exposures, thereby not much accumulation of lipid-laden lysosomes. Nevertheless, further studies on cell-specific changes in senescence and other molecular hallmarks of aging (e.g. mitochondrial dysfunction, telomere shelterin complexes, proteostasis, epigenetics, and molecular clock) associated with molecular imaging by CS in younger and older mice, and human lungs would provide more information on aging and their susceptibility to COPD.

Despite having a high genetic homology between the genomes of mice and humans, the COPD phenotype is different in mice when compared with human. Mice have a restricted number of submucosal glands and possess low number of goblet cells^69^. On exposure to CS, a cluster of goblet cells appear in their bronchi as a result of metaplasia. In addition, mice develop patchy emphysema instead of centrilobular emphysema due to the absence of respiratory bronchioles^69^. Mice are unable to completely recapitulate human disease. Disease phenotype varies with different strains and sexes^70^. Earlier studies suggest that sex is an important determinant of CS-induced extent of emphysema^71,72^. We, however, were unable to differentiate the sex-dependent CS-induced cellular senescence with aging due to an insufficient number of male and female mice. It is therefore important to carry out further studies based upon sexes and strains in order to understand whether sex influences aging phenotype and cellular senescence^70^.

In conclusion, our study demonstrates that chronic CS exposure and aging causes lung cellular senescence by increasing SA-β-gal activity associated with lung function decline, but does not further augment severity of COPD/emphysema in mice. Further, aging actively takes part to increase CS-induced inflammatory response in older mice, whereas levels of DAMPs remain unaffected. We, for the first time, show that aging influences SA-β-gal activity, but does not affect cellular senescence and senescence-associated genes by CS in an age-dependent manner in a mouse model of COPD/emphysema. Overall, lung cellular senescence and other biological hallmarks of lung damage are independent of aging in the CS-induced mouse model of COPD/emphysema. Identifying new genes and related canonical signaling pathways that mediates CS-induced cellular senescence during the pathogenesis of COPD and other specific hallmarks for lung aging (particularly in human lungs) will provide novel insights to devise targeted therapy against smoking-induced premature lung aging in the pathogenesis of COPD/emphysema and other age-related chronic lung diseases.

## Electronic supplementary material

Supplementary Table 1

Supplementary Fig 1
