# Supplementary material for: Lung cellular senescence is independent of aging in a mouse model of COPD/emphysema
Source: Sci Rep. 2018 Jun 13;8:9023. doi: 10.1038/s41598-018-27209-3 (PMC5998122; doi:10.1038/s41598-018-27209-3)
Supplement: Supplementary file 1 — Supplementary Table 1 [file 41598_2018_27209_MOESM1_ESM.pdf]

Supplementary Information File

<u>Supplemental Table 1;</u> NanoString custom probe design

Lung cellular senescence is independent of aging in a mouse model of COPD/emphysema

Kahkashan Rashid, Isaac K Sundar, Janice Gerloff, Dongmei Li, and Irfan Rahman

CODESET DETAILS **TABLE -1**

Sl.No.	Identifier	Accession	Position	Target Sequence	Tm CP	Tm RP	Flags	HUGO Gene	NSID
1	AGER	NM_007425.2	362-461	CAACTACCGAGTCCGAGTCTACCAGATTCCTGGGAAGCCAGAAATTGTGGATCCTGCCTCTGAACTCACAGCCAGTGTCCCTAATAAGGTGGGGACATGT	86	84		Ager	NM_007425.2:361
2	ATM	NM_007499.2	5544-5643	TTCCTCAAAGTGAAAGTCATGACATTTGGATAAAGACACTGACGTGTGCCTTTCTGGACAGTGGAGGCATAAACAGTGAAATTCTCCAGTTATTAAAGCC	81	79		Atm	NM_007499.2:5543
3	BUB1B	NM_009773.3	21-120	CTGCGTCAAGAGACGTTAAATTTGAAACTTGGCGGCCCGTGCGTGATGGGGGCGTGAGGAGGCTATTCTGAGAAGGAATCGGGTGCGTGGTTTTGTTTAG	83	81		Bub1b	NM_009773.3:20
4	CAMP	NM_009921.2	356-455	AACCCGGCCGCTGATTCTTTTGACATCAGCTGTAACGAGCCTGGTGCACAGCCCTTTCGGTTCAAGAAAATTTCCCGGCTGGCTGGACTTCTCCGCAAAG	83	82		Camp	NM_009921.2:355
5	CCL2	NM_011333.3	416-515	TCTTCAGCACCTTTGAATGTGAAGTTGACCCGTAAATCTGAAGCTAATGCATCCACTACCTTTTCCACAACCACCTCAAGCACTTCTGTAGGAGTGACCA	79	82		Ccl2	NM_011333.3:415
6	CDKN1A	NM_007669.4	1671-1770	AATACCGTGGGTGTCAAAGCACTTAGTGGGTCTGACTCCAGCCCCAAACATCCCTGTTTCTGTAACATCCTGGTCTGGACTGTCTACCCTTAGCCCGCAC	82	82		Cdkn1a	NM_007669.4:1670
7	CDKN2A	NM_001040654.1	567-666	CCAATCCCAAGAGCAGAGCTAAATCCGGCCTCAGCCCGCCTTTTTCTTCTTAGCTTCACTTCTAGCGATGCTAGCGTGTCTAGCATGTGGCTTTAAAAAA	83	78		Cdkn2a	NM_001040654.1:566
8	CXCL8	NM_011339.2	420-519	AGAGAATATTTCCCTTTCCAATTCGGGAGACCTCTAGACACTTTGCTGATTTAGCTCACAACAGTGATAGGAATTTTCTACGGGACTCCAGTGAAGTCAG	78	81		Cxcl15	NM_011339.2:419
9	EZH2	NM_007971.2	426-525	AGTCATCCCGTTAAAGACCCTGAATGCAGTCGCCTCGGTGCCTATAATGTACTCTTGGTCGCCCTTACAACAGAATTTTATGGTGGAAGACGAAACTGTT	83	79		Ezh2	NM_007971.2:425
10	FN1	NM_010233.1	2628-2727	TCCAGACCCTACCGTGGACCAGGTTGATGATACTTCCATTGTTGTTCGGTGGAGTAGACCCCAGGCACCTATCACAGGGTATAGAATTGTCTATTCACCT	81	83		Fn1	NM_010233.1:2627
11	GAPDH	NM_001001303.1	891-990	AGGTTGTCTCCTGCGACTTCAACAGCAACTCCCACTCTTCCACCTTCGATGCCGGGGCTGGCATTGCTCTCAATGACAACTTTGTCAAGCTCATTTCCTG	81	79	HK	Gapdh	NM_001001303.1:890
12	H2AFX	NM_010436.2	981-1080	TGGCCTCATACCAGTTGACCCTGTCGCCATTCGTAGGCCAGTGTGGTGTGGCGGGGCCAAAAATGGTTGTAATGTGAACCCAGTTTCTCTAGGAATACCT	82	83		H2afx	NM_010436.2:980
13	HAS2	NM_008216.3	3389-3488	TGTGGCAGACCTTCTCACATGCACAATGAGTGTGTTTCCTCCAGTTAGTGTCTGGGTTCGCCAAGTCACCCTGCCTGTTAGTGATGTTTACATGGGCGAA	80	83		Has2	NM_008216.3:3388
14	HDAC2	NM_008229.2	1011-1110	CTGGGGACAGGCTTGGTTGTTTCAATCTAACTGTCAAAGGTCATGCTAAATGTGTAGAAGTAGTGAAAACTTTTAACTTGCCATTGCTGATGCTCGGTGG	80	78		Hdac2	NM_008229.2:1010
15	HMGB1	NM_010439.3	1575-1674	GTGGGACTATTAGGATCAAGCAATCTGAACGTCTGTCCTTGAAGGACTGATAGAAAAGTACCTTCTAATCCTTACACGAGGACTCTCCTTTAACCGCCAT	84	79		Hmgb1	NM_010439.3:1574
16	HPRT1	NM_013556.2	31-130	TGCTGAGGCGGCGAGGGAGAGCGTTGGGCTTACCTCACTGCTTTCCGGAGCGGTAGCACCTCCTCCGCCGGCTTCCTCCTCAGACCGCTTTTTGCCGCGA	82	82	HK	Hprt	NM_013556.2:30
17	HSPA4	NM_008300.3	4311-4410	TGACTGTCCTTAATGAAGAACTCATAAACATTTGTGTGTGGGGGTGGGGGTGGAGGGTTTGGGTGCTGGGATGGGGGTGAATTTGAAGTTTAAGATTAA	82	82		Hspa4	NM_008300.3:4310
18	IGF1	NM_001111274.1	419-518	GGGCTTTTACTTCAACAAGCCCACAGGCTATGGCTCCAGCATTCGGAGGGCACCTCAGACAGGCATTGTGGATGAGTGTTGCTTCCGGAGCTGTGATCTG	83	84		Igf1	NM_001111274.1:418
19	IL1B	NM_008361.3	1121-1220	GTTGATTCAAGGGGACATTAGGCAGCACTCTCTAGAACAGAACCTAGCTGTCAACGTGTGGGGGATGAATTGGTCATAGCCCGCACTGAGGTCTTTCATT	82	80		Il1b	NM_008361.3:1120
20	IL6	NM_031168.1	41-140	CTCTCTGCAAGAGACTTCCATCCAGTTGCCTTCTTGGGACTGATGCTGGTGACAACCACGGCCTTCCCTACTTCACAAGTCCGGAGAGGAGACTTCACAG	82	82		Il6	NM_031168.1:40
21	KL	NM_013823.2	1165-1264	CTGATTTTACTGAATCTGAGAAGAGGCTCATCAGAGGAACTGCTGACTTTTTTGCTCTCTCCTTCGGACCAACCTTGAGCTTTCAGCTATTGGACCCTAA	81	80		Kl	NM_013823.2:1164
22	LMNB1	NM_010721.2	806-905	ATCAGATTGCCCAGCTAGAAGCATCCTTATCTGCCGCCAAAAAGCAGTTAGCAGATGAAACTTTACTTAAAGTGGATTTGGAGAATCGCTGTCAGAGCCT	83	80		Lmnb1	NM_010721.2:805
23	MMP12	NM_008605.3	593-692	AAGGTGGTACACTAGCCCATGCTTTTTATCCTGGACCTGGTATTCAAGGAGATGCACATTTTGATGAGGCAGAAACGTGGACTAAAAGTTTTCAAGGCAC	82	80		Mmp12	NM_008605.3:592
24	MMP9	NM_013599.2	1571-1670	CCTCTACAGAGTCTTTGAGTCCGGCAGACAATCCTTGCAATGTGGATGTTTTTGATGCTATTGCTGAGATCCAGGGCGCTCTGCATTTCTTCAAGGACGG	78	80		Mmp9	NM_013599.2:1570
25	MTOR	NM_020009.2	2433-2532	CATGGAGCCTATCCTGAAGGCTTTAATTTTGAAACTGAAAGATCCAGACCCTGACCCAAACCCGGGCGTGATCAATAACGTGTTGGCCACTATAGGAGAA	80	84		Mtor	NM_020009.2:2432
26	PCNA	NM_011045.2	591-690	AGACCTTAGCCACATTGGAGATGCTGTTGTGATATCCTGTGCAAAGAATGGGGTGAAGTTTTCTGCAAGTGGAGAGCTTGGCAATGGGAACATTAAGTTG	82	81		Pcna	NM_011045.2:590
27	PIK3CA	NM_008839.1	1256-1355	ACTGTCCGTTGGCCTGGGGAAACATAAACTTGTTTGATTATACAGACACCCTAGTGTCCGGGAAAATGGCTTTGAATCTCTGGCCTGTACCGCATGGGTT	81	82		Pik3ca	NM_008839.1:1255
28	PPARGC1	NM_008904.2	691-790	CGAGAATTCATGGAGCAATAAAGCGAAGAGCATTTGTCAACAGCAAAAGCCACAAAGACGTCCCTGCTCAGAGCTTCTCAAGTATCTGACCACAAACGAT	81	82		Ppargc1a	NM_008904.2:690
29	PRKAA2	NM_178143.1	1891-1990	TCCAGCATTCTGTTTACTCGTAGAACTCTGTAACTCTCCTGTGCCTGTGACAAGTTTGCATGGGTAGTACTGGGTGAGTGTGGTGGCGCTTGCTAACTTA	79	83		Prkaa2	NM_178143.1:1890
30	RGN	NM_009060.2	83-182	TCAAAGTTGAATGTGTTTTACGGGAGAACTACAGGTGTGGGGAGTCTCCTGTATGGGAGGAAGCGTCACAGTCGCTACTGTTTGTAGATATCCCTTCAAA	82	80		Rgn	NM_009060.2:82
31	RPL19	NM_009078.1	21-120	GAAGAGGCTTGCCTCTAGTGTCCTCCGCTGCGGGAAAAAGAAGGTCTGGTTGGATCCCAATGAGACCAATGAAATCGCCAATGCCAACTCCCGTCAGCAG	83	80	HK	Rpl19	NM_009078.1:20
32	S100A8	NM_013650.2	281-380	ATAAAAGTGGGTGTGGCATCTCACAAAGACAGCCACAAGGAGTAGCAGAGCTTCTGGCCTAGGGCTGGGTCCCTGGATATGTCTACAGAATAAAGTCATC	82	82		S100a8	NM_013650.2:280
33	SERPINB2	NM_001174170.1	716-815	TGGGCTTTATCCTTTCCGTGTGAACTCGCATGAGAGCATACCTGTCCAGATGATGTTCCTCCATGCAAAGCTGAACATTGGATACATAAAGGACCTGAAG	82	82		Serpinb2	NM_001174170.1:715
34	SERPINE1	NM_008871.2	1823-1922	AGGGGCAACGGATAGACAGATCAAATGGTGGCCCAATAGCGAGCCTTCTCCCTGCTCCCTCCCTTGACACAGCTTGCTTATGTTATTTCAGAGTGTAGGT	84	79		Serpine1	NM_008871.2:1822
35	SHC1	NM_001113331.2	1957-2056	ATCGGAAAGTGTGATCCTTCTCAGCTTCTCCAACAGGATGCTCTCCATTTCCGTCTCCCGTATTCTCTAACTTGTGGGACCTCTGTTTTGTGGGTCTGGC	79	80		Shc1	NM_001113331.2:1956
36	SIRT1	NM_019812.2	844-943	GGATTCCTGACTTCAGATCAAGAGACGGTATCTATGCTCGCCTTGCGGTGGACTTCCCAGACCTCCCAGACCCTCAAGCCATGTTTGATATTGAGTATTT	83	79		Sirt1	NM_019812.2:843
37	TBP	NM_013684.3	71-170	GTGGCGGGTATCTGCTGGCGGTTTGGCTAGGTTTCTGCGGTCGCGTCATTTTCTCCGCAGTGCCCAGCATCACTATTTCATGGTGTGTGAAGATAACCCA	83	79	HK	Tbp	NM_013684.3:70
38	TERC	NR_001579.1	55-154	TGTTTTTCTCGCTGACTTCCAGCGGGCCAGGAAAGTCCAGACCTGCAGCGGGCCACCGCGCGTTCCCGAGCCTCAAAAACAAACGTCAGCGCAGGAGCTC	86	89		Terc	NR_001579.1:54
39	TERF2	NM_001083118.2	773-872	AAAAGAACTTGGCCCACCCTGTTATCCAGAACTTTTCCTATGAGGTCTTCCAGCAGAAGATGCTGCGTTTCCTAGAGAGCCACCTGGATGACACGGAGCC	79	83		Terf2	NM_001083118.2:772
40	TERT	NM_009354.1	2163-2262	CAGGGTAAGCTGGTGGAGGTTGTTGCCAATATGATCAGGCACTCGGAGAGCACGTACTGTATCCGCCAGTATGCAGTGGTCCGGAGAGATAGCCAAGGCC	83	82		Tert	NM_009354.1:2162
41	TGFB1	NM_011577.1	1471-1570	GGAGTTGTACGGCAGTGGCTGAACCAAGGAGACGGAATACAGGGCTTTCGATTCAGCGCTCACTGCTCTTGTGACAGCAAAGATAACAAACTCCACGTGG	82	78		Tgfb1	NM_011577.1:1470
42	TP53	NM_011640.1	1836-1935	CCCTCTCTGAGTAGTGGTTCCTGGCCCAAGTTGGGGAATAGGTTGATAGTTGTCAGGTCTCTGCTGGCCCAGCGAAATTCTATCCAGCCAGTTGTTGGAC	82	83		Trp53	NM_011640.1:1835
43	TP53BP1	NM_013735.3	3795-3894	TGGCCATGTCTTGCATCGCCACATGAGAACCATTCGAGAAGTCCGTACACTCGTCACCCGCGTCATCACAGATGTTTATTATGTGGATGGGACAGAAGTG	81	80		Trp53bp1	NM_013735.3:3794
44	TUBB5	NM_011655.4	2261-2360	ATTGGAAGTGTCTTCCCTGTATTGGTTCTCCTTTCTCGGAGAGATGGGGGTTGGGGGTGCGGCAAGGTCTTGGTCTTGGTCTCTGAACACTCCCAATTCC	79	82	HK	Tubb5	NM_011655.4:2260
45	WRN	NM_001122822.1	1903-2002	TAGGGTCATCTACATAACTCCAGAGTTCTGTTCTGGTAACTTGGATCTACTCCAGCAACTTGACTCTAGTATTGGCATCACTCTCATTGCTGTGGATGAG	79	82		Wrn	NM_001122822.1:1902
46	ZMPSTE24	NM_172700.2	1435-1534	ACCTTACAATGAGGTTCTGTCTTTCTGCCTAACAGTCCTGAGCCGCAGATTTGAATTTCAAGCTGATGCGTTTGCCAAGAAACTTGGGAAGGCTAAAGAC	82	81		Zmpste24	NM_172700.2:1434
