# Supplementary material for: Lung cellular senescence is independent of aging in a mouse model of COPD/emphysema
Source: Sci Rep. 2018 Jun 13;8:9023. doi: 10.1038/s41598-018-27209-3 (PMC5998122; doi:10.1038/s41598-018-27209-3)

Supplementary Information File

Lung cellular senescence is independent of aging in a mouse model of COPD/emphysema

Kahkashan Rashid, Isaac K Sundar, Janice Gerloff, Dongmei Li, and
Irfan Rahman

Full unedited gel for Fig. 1(B) p16 and GAPDH

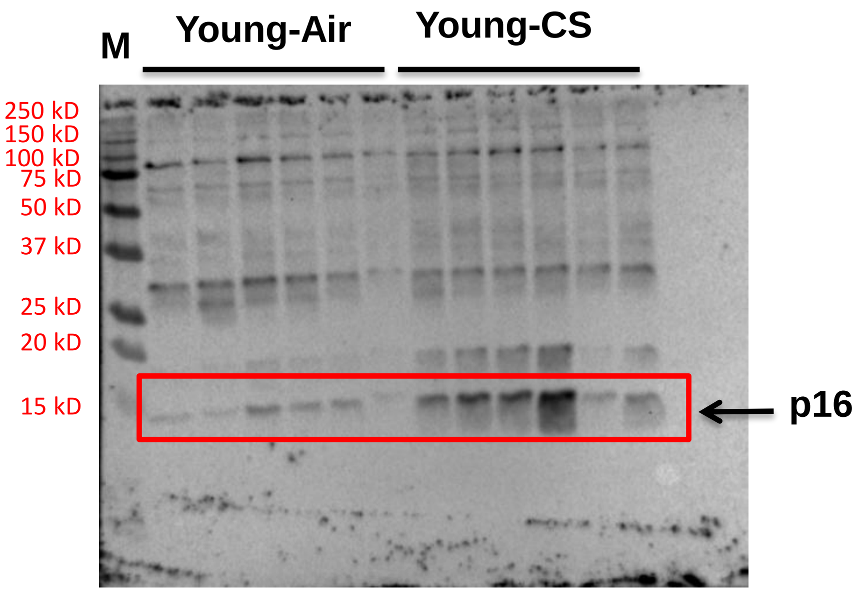

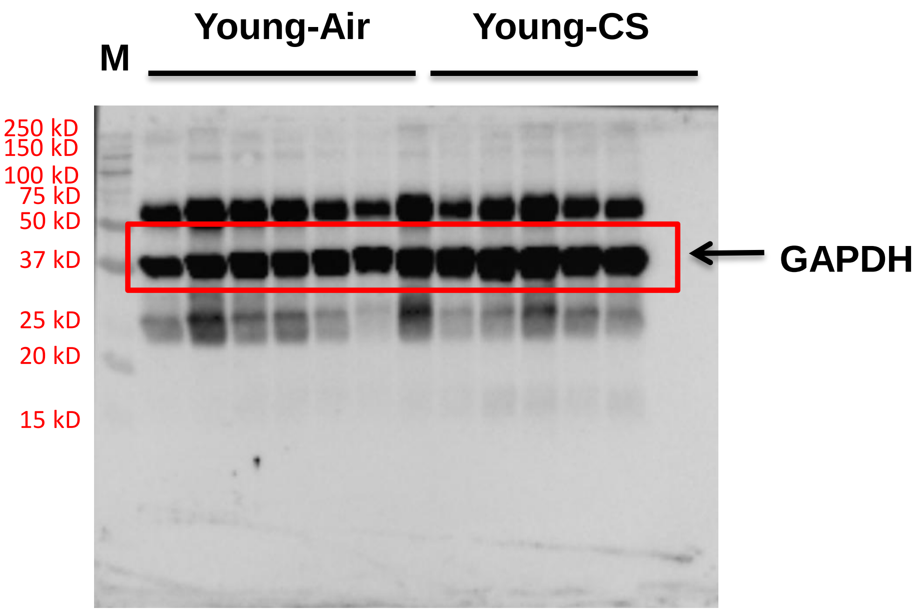

Full unedited gel for Fig. 1(B) p16 and Actin

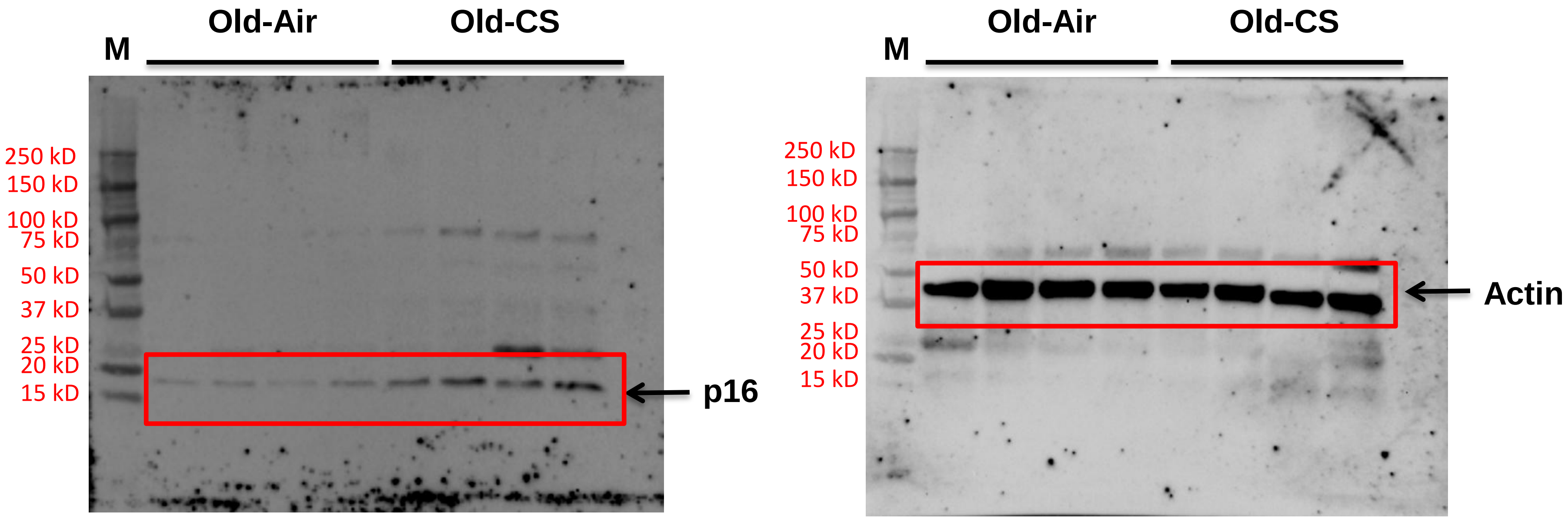

Supplement: Supplementary file 2 — Supplementary Fig 1 [file 41598_2018_27209_MOESM2_ESM.pdf]
